# The Opposing Contribution of SMS1 and SMS2 to Glioma Progression and Their Value in the Therapeutic Response to 2OHOA

**DOI:** 10.3390/cancers11010088

**Published:** 2019-01-14

**Authors:** Paula Fernández-García, Catalina A. Rosselló, Raquel Rodríguez-Lorca, Roberto Beteta-Göbel, Javier Fernández-Díaz, Victoria Lladó, Xavier Busquets, Pablo V. Escribá

**Affiliations:** 1 Laboratory of Molecular Cell Biomedicine, University of the Balearic Islands, 07122 Palma de Mallorca, Spain; ca.rossello@lipopharma.com (C.A.R.); raquel.rodriguez@uib.es (R.R.-L.); roberto.beteta@uib.es (R.B.-G.); j.fernandez@uib.es (J.F.-D.); Victoria.llado@uib.es (V.L.); xavier.busquets@uib.es (X.B.); pablo.escriba@uib.es (P.V.E.); 2 Lipopharma Therapeutics, Isaac Newton, 07121 Palma de Mallorca, Spain

**Keywords:** SMS1, SMS2, glioma, biomarker, 2OHOA

## Abstract

*Background*: 2-Hydroxyoleic acid (2OHOA) is particularly active against glioblastoma multiforme (GBM) and successfully finished a phase I/IIA trial in patients with glioma and other advanced solid tumors. However, its mechanism of action is not fully known. *Methods*: The relationship between SMS1 and SMS2 expressions (mRNA) and overall survival in 329 glioma patients was investigated, and so was the correlation between SMS expression and 2OHOA’s efficacy. The opposing role of SMS isoforms in 2OHOA’s mechanism of action and in GBM cell growth, differentiation and death, was studied overexpressing or silencing them in human GBM cells. *Results*: Patients with high-SMS1 plus low-SMS2 expression had a 5-year survival ~10-fold higher than patients with low-SMS1 plus high-SMS2 expression. SMS1 and SMS2 also had opposing effect on GBM cell survival and 2OHOA’s IC_50_ correlated with basal SMS1 levels and treatment induced changes in SMS1/SMS2 ratio. SMSs expression disparately affected 2OHOA’s cancer cell proliferation, differentiation, ER-stress and autophagy. *Conclusions*: SMS1 and SMS2 showed opposite associations with glioma patient survival, glioma cell growth and response to 2OHOA treatment. SMSs signature could constitute a valuable prognostic biomarker, with high SMS1 and low SMS2 being a better disease prognosis. Additionally, low basal SMS1 mRNA levels predict positive response to 2OHOA.

## 1. Introduction

Glioblastoma multiforme (GBM), the most malignant type of glioma, is the most common type of brain tumor, and it is associated with a low median survival of 9–15 months [1]. The bioactive lipid molecule, 2OHOA, was shown to be safe in patients with glioma and other advanced solid tumors in a phase I/IIa clinical trial (ClinicalTrials.gov identifier #NCT01792310). In a xenograft model of human GBM, 2OHOA provided a greater anti-tumor effect than temozolomide (TMZ), which is the current standard-of-care first-line chemotherapy against GBM and increases patient’s survival by ca. 2.5 months [2,3]. In mice, tumors relapsed when treated with TMZ, but not in mice treated with 2OHOA. 

2OHOA is a hydroxylated fatty acid that produces dramatic changes in membrane lipid composition and organization [4,5], activating sphingomyelin (SM) synthesis [6]. Sphingomyelin synthase (SMS) is an enzyme that generates SM. The two main SMS isoforms, SMS1 and SMS2, share significant homology, except for a sterile alpha motif (SAM), present only in SMS1 (Figure S1), which can interact with regulatory proteins, lipids and RNA [7]. In addition, the SMS isoforms differ in their subcellular localization: SMS1 is located in the Golgi apparatus, and SMS2 in the Golgi apparatus and the plasma membrane [8]. Most SM is synthetized by SMS1, which is responsible for the 60–80% of the SMS activity [9,10]. SMSs can reversibly catalyze SM synthesis by transferring phosphocholine or phosphoethanolamine from phosphatidylcholine or phosphatidylethanolamine onto ceramide, yielding SM and diacylglycerol [11,12]. Ceramide and diacylglycerol are widely known for their pro- and anti-apoptotic roles, respectively [13,14]. In addition, SM and cholesterol are the main components of lipid rafts, specialized regions of the plasma membrane with important roles in signal transduction and membrane trafficking [15,16]. Therefore, regulation of SM by 2OHOA has relevant consequences for cancer cell signaling and may explain 2OHOA antitumor pharmacological effects. Neither the role of SMS1 and SMS2 in tumor progression nor the effect of 2OHOA on each SMS isozymes are fully understood.

A useful tool to identify potential pharmacological targets for brain tumors is the REpository for Molecular BRAin Neoplasia DaTa (REMBRANDT) database, which integrates information on gene expression and survival [17]. Because 2OHOA has relevant antitumor activity against glioma, and given that its mechanism of action involves SMS activation and altered SMS1 expression is associated with glioma patient survival, we studied the role of these enzymes in glioma tumorigenesis, prognosis and response to 2OHOA [3,18,19]. In this study, we found that SMS1 and SMS2 expression had an opposing impact on patient survival, which is in line with their divergent effects on GBM cell survival and is consistent with 2OHOA-induced activation of SMS1. Whether both SMS isozymes catalyze similar reactions or differ in their activity in resting and growing (e.g., cancer) cells requires additional investigation. The present study further demonstrates that SMS isoforms are valuable anticancer drug targets and tumor biomarkers.

## 2. Results

### 2.1. SMS1 and SMS2 Expression Have Opposite Effects on Glioma Patient Survival

Using the REMBRANDT database, we investigated the expression of SMS1 and SMS2 in all the types of glioma. BETASTASIS website provides gene expression and survival data from 329 glioma patients, of which 54 patients were alive, and therefore censored and only used for expression analyses, and not in survival studies. Kaplan-Meier plots were used to assess the median survival for patient groups with differential SMS1 or SMS2 gene expression and significant differences in survival (*p* < 0.05) were determined using the log-rank test.

**Figure 1:**
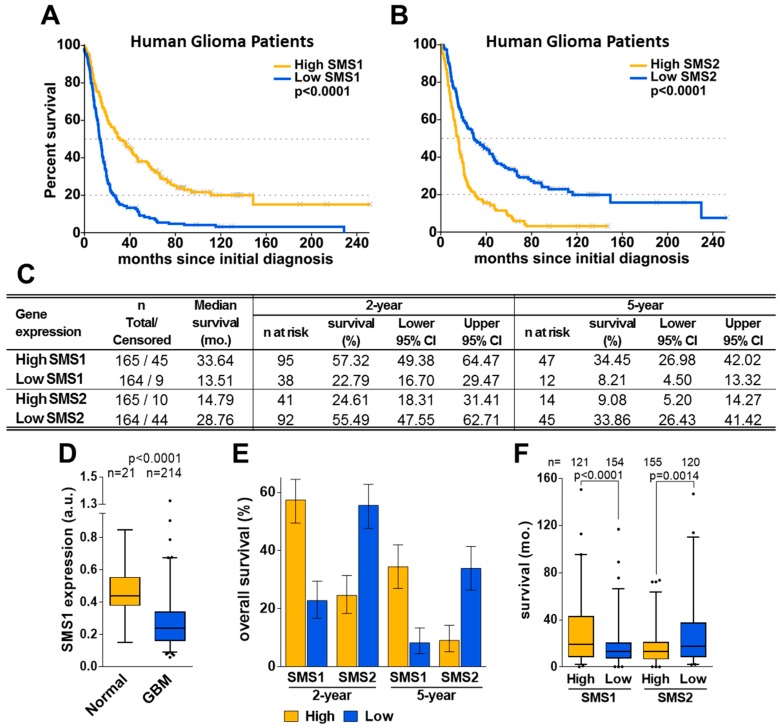
SMS1 and SMS2 expression and glioma patient survival. Kaplan-Meier plots showing the overall survival of glioma patients (from the REMBRANDT database) as a function of SMS1 (**A**) or SMS2 (**B**) expression. (**C**) Survival parameters (mo.: months; n: number of patients; CI: confidence interval). (**D**) SMS1 expression comparison in brain tissue from healthy subjects (Normal) and GBM patients. (**E**) Two- and five-year survival of glioma patients with high (orange) or low (blue) SMS1 and SMS2 expression. (**F**) Patient’s survival (months) depending on Low/High SMS1 (left) or SMS2 (right) expression (censored events, corresponding to alive patients, were excluded). The differences between groups were analyzed using a non-parametric Mann-Whitney test.

When analyzing SMS1 and SMS2 gene expression, glioma patients were classified into 2 groups defined by high or low SMS1 or SMS2 expression with respect to the median expression for each gene. Patients with high SMS1 expression (SMS1-high) had longer median survival than patients with low SMS1 expression (SMS1-low; Figure 1A,C). Compared to normal tissue from healthy subjects, SMS1 had a significantly lower expression in GBM patients (Figure 1D), thus indicating its implication in tumorigenesis and its value as a prognostic factor. Conversely, patients with low SMS2 expression had longer median survival than patients with high SMS2 expression (Figure 1B,C). Consistent with the median survival, the two-year and five-year survival rates were higher for SMS1-high or SMS2-low groups, whereas patients in SMS1-low or SMS2-high groups had a worse prognosis (Figure 1 C,E,F). 

**Figure 2:**
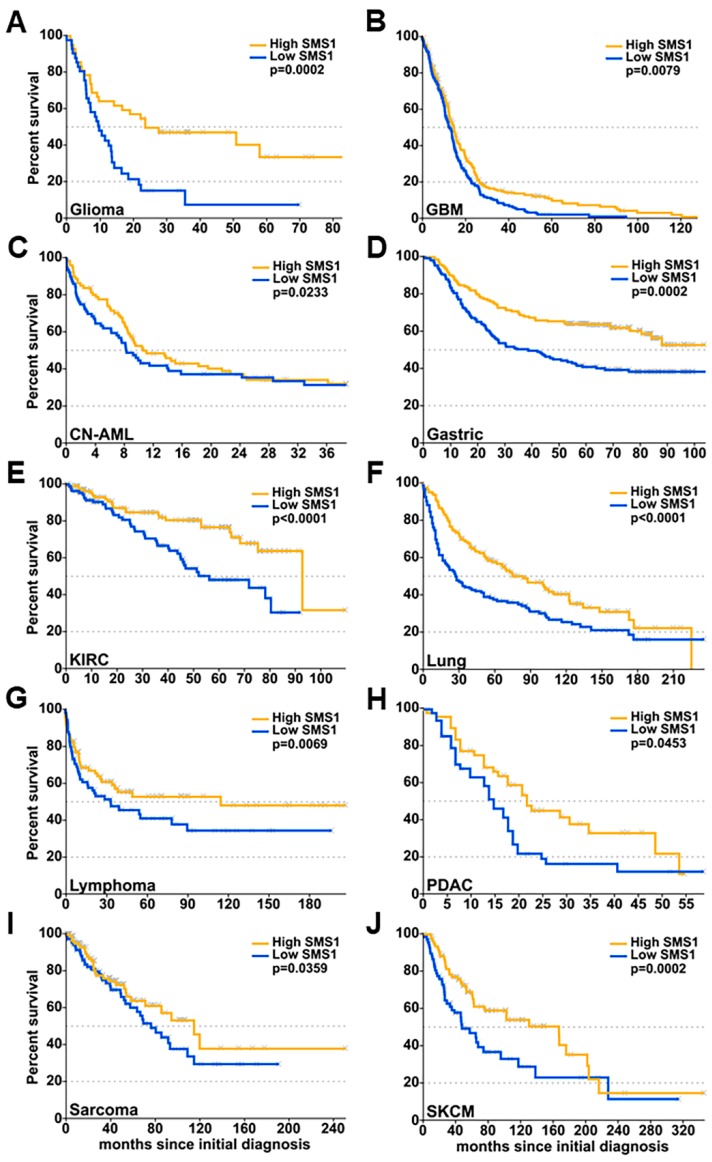
SMS1 expression and overall survival in patients with different types of cancer. Kaplan-Meier plots representing overall survival as a function of SMS1 expression measured in biopsies from patients with (**A**) glioma (grade III and IV, from the GSE4412 database; *n* = 83), (**B**) glioblastoma multiforme (GBM; *n* = 542), (**C**) cytogenetically normal acute myeloid leukemia (CN-AML; *n* = 163), (**D**) gastric cancer (*n* = 299), (**E**) kidney renal clear cell carcinoma (KIRC; *n* = 234), (**F**) lung cancer (*n* = 282), including NSCLC (non-small cell lung cancer) and SCLC (small cell lung cancer), (**G**) lymphoma (B-cell lymphoma and Burkitt’s Lymphoma; *n* = 158), (**H**) pancreatic ductal adenocarcinoma (PDAC; *n* = 102), (**I**) sarcoma (*n* = 234), or (**J**) skin cutaneous melanoma (SKCM; *n* = 163). For each data set, patients’ cancer samples were classified as high- and low-expressing tissue if the SMS1 expression was higher or lower than the median expression. The *p* values from the log-rank tests comparing the two curves are shown in each figure. n: number of patients in each data set.

The importance of SMS1 expression for patient survival was confirmed in other databases for glioma (GSE4412 database, Figure 2A) and GBM (TCGA database, Figure 2B) and in other cancer types analyzed: Normal acute myeloid leukemia (CN-AML, Figure 2C), gastric cancer (Figure 2D), kidney renal clear cell carcinoma (KIRC, Figure 2E), lung cancer, including NSCLC (non-small cell lung cancer) and SCLC (small cell lung cancer) (Figure 2F), lymphoma (B-cell lymphoma and Burkitt’s Lymphoma, Figure 2G), pancreatic ductal adenocarcinoma (PDAC, Figure 2H), sarcoma (Figure 2I) and skin cutaneous melanoma (SKCM, Figure 2J) (Table 1). Despite similar enzymatic activity, SMS1 and SMS2 have a divergent influence on glioma patients’ overall survival, and this divergence is possibly associated with the different roles in cancer (and possibly normal) cell differentiation, proliferation and survival.

### 2.2. SMS1/SMS2 Ratio is Relevant for Glioma Patient Survival and Correlates with 2OHOA’s Pharmacological Efficacy

**Figure 3:**
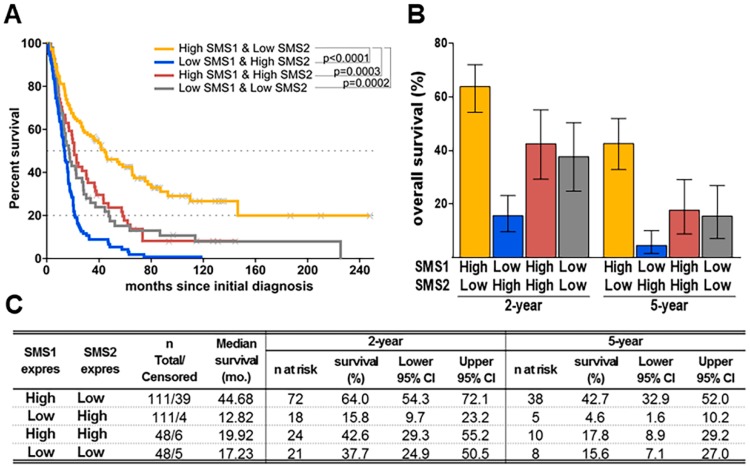
SMS1-to-SMS2 ratio relative to patient survival. (**A**) Kaplan-Meier plots representing glioma patient survival depending on SMS1 and SMS2 combined expressions. Patients’ biopsy samples were classified into four groups depending, firstly, on their SMS1 (considering high-expressing group when SMS1 expression was equal or higher than the median) and, secondly, on their SMS2 expression (SMS1 low and high groups were divided considering SMS2 expression median from all samples). (**B**) Overall-2-, and -5-year survivals after diagnostic in glioma patients (REMBRANDT database) classified according to their SMS1 and SMS2 combined expressions. Data are presented as Mean ± Error. (**C**) Patients at risk and median survival determined from glioma patients’ dataset as represented in (**A**); mo.: months; n: number of patients; CI: confidence interval.

To evaluate the combined influence of SMS1 plus SMS2 gene expression in glioma patients, 4 groups were evaluated (SMS1-high plus SMS2-low, SMS1-low plus SMS2-low, SMS1-high plus SMS2-high and SMS1-low plus SMS2-high), and significant differences were observed (Figure 3A). SMS1-high plus SMS2-low group had a 5-year survival of 42.7%, whereas SMS1-low plus SMS2-high group had a 5-year survival of 4.6% (Figure 3B,C). These were the best and worst median survival groups, respectively, whereas the other 2 groups displayed an intermediate median survival.

**Figure 4:**
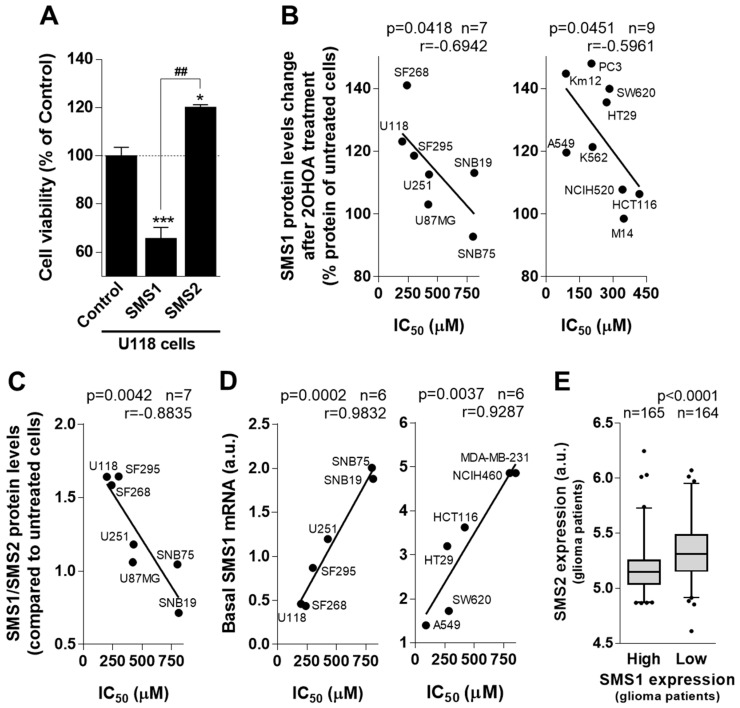
SMS1 expression and protein levels correlate with 2OHOA’s pharmacological potency. (**A**) Viability of human GBM (U118) cells overexpressing SMS1 or SMS2 relative to control cells (100% viability). (**B**) Correlation between the changes in SMS1 protein after 2OHOA treatment (48 h; % of protein levels compared to untreated cells, 100%) and the IC_50_ values in seven glioma cancer cell lines (left) or nine non-glioma cancer cell lines (right). (**C**) Correlation between changes in the SMS1:SMS2 protein ratio, determined by immunoblotting, after 2OHOA treatment (48 h) with respect to untreated cells (SMS1/SMS2 = 1) and the IC_50_ values in various human GBM cell lines. (**D**) Correlation between SMS1 mRNA basal levels and IC_50_ values for 2OHOA in six glioma cell lines (left) or in six non-glioma cancer cell lines (right). Relative amount of basal mRNA was obtained by quantitative reverse-transcriptase polymerase chain reaction (RTqPCR), according to a standard curve in which different amounts of control mRNA were amplified for more precise quantification. Correlations were analyzed using the Pearson’s correlation coefficient (r) and considered significant when *p* < 0.05. In all panels, n indicates the number of cell lines included in the analysis (**B**–**D**) or the number of patient samples analyzed (**E**). Every point in correlations correspond to mean values of 2–4 independent experiments. (**E**) SMS2 expression in patients with low (*n* = 164) or high (*n* = 165) SMS1 expression levels. Data from the REMBRANDT database.

To assess the functional relevance of the previous findings in patients, in vitro cultures of several glioma and other tumor cell lines were used. To investigate the divergent effects of SMS isoforms on human GBM (U118) cell proliferation, gain-of-function assays for SMS1 and SMS2 were performed. To this end, cells were transfected with the Tet-On/Off 3G system containing either SMS1 or SMS2 cDNA, which induced SMS isozyme overexpression upon doxycycline addition to the culture medium (Figures S2 and S3). U118 cells overexpressing SMS1 showed a 35% lower viability than non-induced control cells. In contrast, cells overexpressing SMS2 showed higher viability (20%) than control GBM cells (Figure 4A). Human GBM (SF295) cells exhibited a similar viability behavior after overexpression of SMS1 and SMS2 (Figure S4). These results indicated that SMS1 and SMS2 fulfill different roles in glioma cell growth and possibly in tumorigenesis. 

Loss-of-function experiments were carried out inducing 48-h SMS1 and SMS2 silencing by means of specific siRNAs. In both cases, compromised cell viability was observed, which is in agreement with the proliferative role of SMS2 (−89.7% and −48.1%, respectively, Figure S5). The effect observed for SMS1 could be due to the high level of silencing achieved as discussed below.

In addition, SMS1 and SMS2 protein levels were evaluated in several cancer cell types after exposure to 2OHOA. SMS1 and SMS2 were measured after 48 h in the presence or absence (control, 100%) of 2OHOA (200 µM) in 16 cancer cell lines (Figure 4 and Figure S6), and the IC_50_ for 2OHOA was also determined in these cell lines (Table S1). An inverse correlation was observed between the change in SMS1 protein levels after 2OHOA treatment and their IC_50_ values in 7 human GBM cell lines (Figure 4B left panel). Similar results were obtained when this correlation was assessed in another 9 non-glioma cancer cell lines (Figure 4B right panel). 2OHOA-mediated increase in SMS1 expression correlated with lower IC_50_ values (i.e., stronger antitumor effect). In addition, cell lines with higher increase in SMS1 expression after 2OHOA treatment displayed the lowest basal SMS1 mRNA expression levels (Figure 4D). Moreover, SMS1/SMS2 ratio inversely correlated with IC_50_ values for 2OHOA (Figure 4C and Table S2). These results indicated that glioma cell lines responded better to drug treatment upon SMS1 increase, SMS2 decrease or both. Accordingly, the ratio of these two proteins represents a relevant predictor of drug potency and efficacy. Indeed, an inverse correlation between SMS1 and SMS2 expression was observed when their mRNA levels were analyzed in 386 glioma patients (*p* < 0.0001; Figure 4E), thus further supporting the clinical relevance of the SMS1/SMS2 ratio in glioma progression and prognosis.

### 2.3. Basal SMS1 mRNA Levels Anticipate 2OHOA Anti-Cancer Activity against GBM

Basal SMS1 and SMS2 mRNA levels were evaluated in 6 human glioma cell lines, four responders and two non-responders (IC_50_ > 600 µM) to 2OHOA treatment and in one non-tumor cell line, (MRC-5). In these cell lines, the mRNA levels of SMS1 but not of SMS2 strongly correlated with the IC_50_ values for 2OHOA (Figure 4D left panel) and similar results were observed in other cancer cell lines (Figure 4D right panel). These results suggest that basal SMS1 mRNA levels anticipate whether 2OHOA treatment might be efficacious. Moreover, all GBM cell lines investigated had lower SMS1 expression values (0.2–4.5 a.u.) than non-cancer MRC5 cells (7.5 a.u.: Table S3), similarly as observed in human brain tissue from glioma patients or healthy subjects (Figure 1D). 

### 2.4. Opposing Influence of SMS1 and SMS2 on 2OHOA Regulation of GBM Cell Proliferation and Differentiation

**Figure 5:**
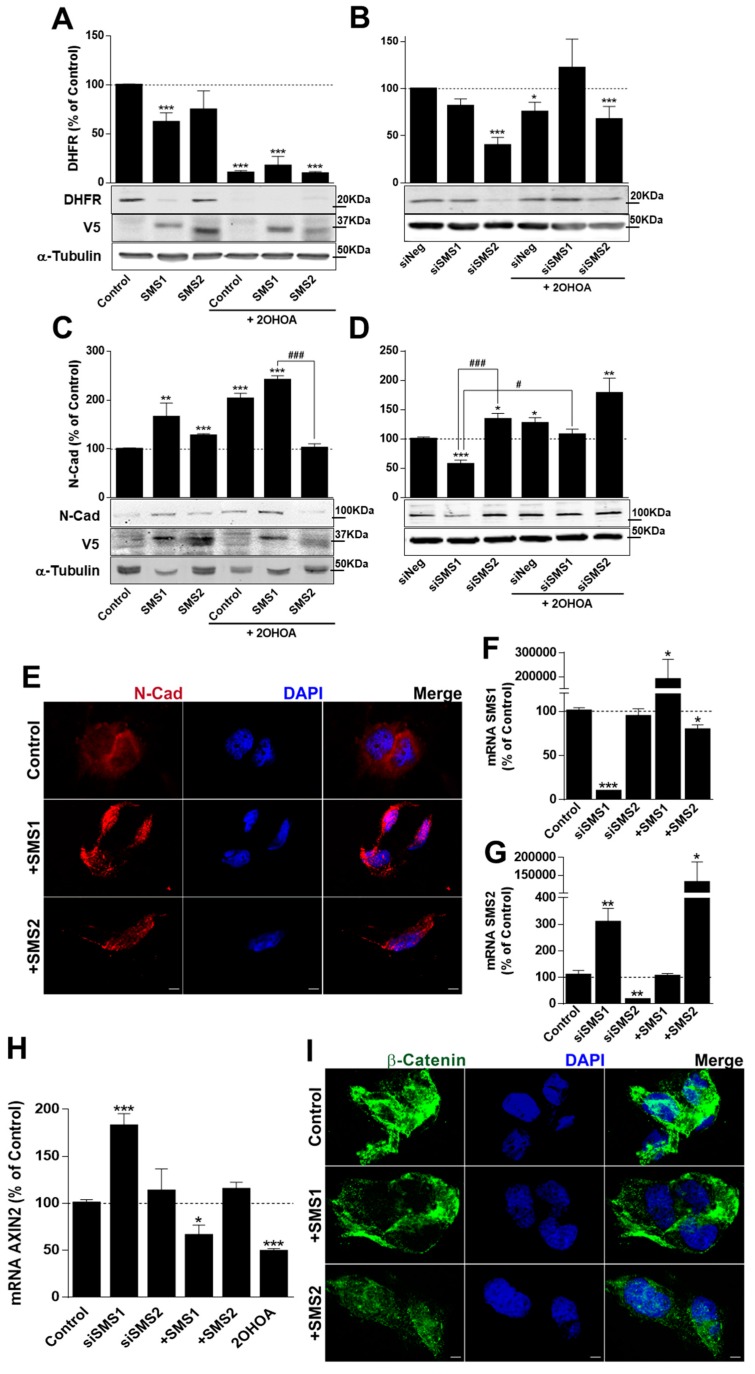
SMSs overexpression and 2OHOA effect on Proliferation and Differentiation in U118 cells. DHFR levels were determined by immunoblotting in U118 cells (**A**) overexpressing SMS1 or SMS2 (Tet-On Inducible Espression System) 72 h after doxycycline administration (1 µg/mL to induce overexpression), exposure to 2OHOA (200 µM) or both, or (**B**) transiently transfected with siRNA against SMS1 or SMS2 for 48 h, after exposure to 2OHOA (200 µM) for 24 h or both. N-Cad levels were determined by immunoblotting in U118 cells (**C**) overexpressing SMS1 or SMS2 (Tet-On Inducible Espression System) 72 h after doxycycline administration (1 µg/mL to induce overexpression), exposure to 2OHOA (200 µM) or both, or (**D**) transiently transfected with siRNA against SMS1 or SMS2 for 48 h, after exposure to 2OHOA (200 µM) for 24 h or both. The values in every lane were normalized to tubulin content and normalized values in treated cells were referred to those of control (untreated and not overexpressing) cells. SMS1 and SMS2 overexpression was measured using an anti-V5 antibody, and the downregulation after siRNA treatment was measured by RTqPCR (**F**,**G**). (**E**) N-Cadherin immunofluorescence (red, confocal microscopy) in U118 cells overexpressing SMS1 or SMS2 for 72 h. SMS1 (**F**) and SMS2 (**G**) mRNA level after 48-h treatment with a non-specific siRNA (Control), siRNA against the SMSs (siSMS1 and siSMS2) for 48 h, and SMSs overexpression (SMS1 and SMS2) for 72 h. (**H**) AXIN2 expression in U118 cells incubated with siSMS1 or siSMS2 for 48 h, overexpressing SMS1 or SMS2 (+SMS1/2), or treated with 2OHOA for 72 h, with respect to untreated cells (Control, 100%). (**I**) β-Catenin immunofluorescence (green) in U118 cells overexpressing SMS1 or SMS2 for 72 h. Nuclei were labeled with DAPI. Representative micrographies (single confocal planes) are shown. Scale bar, 5 µm. All bars represent mean ± s.e.m. values from 2–8 independent experiments; The differences were analyzed using a Student’s *t*-test or ANOVA; *, *p* < 0.05; **, *p* < 0.01; ***, *p* < 0.001; # *p* < 0.05; ###, *p* < 0.001 between the groups indicated.

To evaluate the implication of SMS1 and SMS2 on 2OHOA’s pharmacological mechanism of action, and their differential role on cell viability, overexpression and silencing experiments were carried out on U118 cells in the presence (treated) or absence (control) of 2OHOA. With this aim, we analyzed the relevance of these isoforms in known events of 2OHOA’s mechanism of action. Exposure to 2OHOA induced a significant decrease of dihydrofolate reductase (DHFR) levels in several cancer cell types, including U118 glioma cells, (Figure 5A) [19,20]. The proliferation biomarker (DHFR) decreased 38% when SMS1 was overexpressed and a further decrease to 90% was evident in the presence of 2OHOA (Figure 5A). However, siRNA-mediated SMS1 downregulation had no significant effect on DHFR levels (Figure 5B). In contrast, SMS2 overexpression had no significant effect on DHFR in glioma (U118) cells (Figure 5A), and it produced a significant DHFR increase in SF295 and U251 cells (Figure S7). Conversely, a 70% decrease in DHFR was observed after siRNA-mediated SMS2 downregulation in U118 cells (Figure 5B). It is possible that the effect of 2OHOA on DHFR levels masked any possible effect due to SMSs overexpression. In line with these results, SMS1 silencing prevented 2OHOA-induced DHFR reduction, while SMS2 silencing had no effect on 2OHOA-induced decrease of DHFR levels. These results indicate that 2OHOA-induced activation of SMS1 mediates the ensuing decrease in DHFR levels.

The increase of N-Cadherin inversely correlates with glioma cell invasive capacity and malignant behavior [21], and this increase is associated with glioma cell differentiation [22]. In this context, N-Cadherin levels increased 65% and 30% upon SMS1 or SMS2 overexpression respectively, although a distinct N-Cadherin cellular distribution pattern was observed by confocal microscopy depending on the isoform overexpressed (Figure 5C,E). A 2-fold increase in N-Cadherin was observed after 2OHOA treatment, an effect that was maintained when SMS1 was overexpressed but was abolished when SMS2 was overexpressed (Figure 5C). Accordingly, following siRNA-mediated SMS1 silencing there was a 45% decrease in N-Cadherin, whereas SMS2 downregulation caused a 30% increase in the levels of this protein (Figure 5D). Moreover, silencing of SMS1, but not SMS2, abolished 2OHOA’s effect on N-Cadherin expression. These results further demonstrate the opposing influence of both SMS isoforms on cell proliferation, differentiation and the cellular response to 2OHOA.

Numerous studies have previously shown that alterations in the expression of a receptor subtype or isozyme of a given family can be compensated by the expression of another isoform. To address this possibility, levels of endogenous SMS1 and SMS2 mRNA resulting from different silencing or overexpression technics were evaluated (Figure 5F,G). RNA silencing of SMS1 caused an increase in the expression of SMS2 mRNA levels, but the reverse situation did not occur, silencing of SMS2 did not change SMS1 expression. Interestingly, SMS2 overexpression led to a decrease in SMS1 expression, while increasing SMS1 expression had no effect on SMS2 expression. However, siRNAs treatments did not result in a strong decrease of SMS protein levels, suggesting that small changes in protein amount were enough to produce these effects, or that the protein present was not fully active. The lack of correlation between mRNA levels (Figure 5F,G) and protein levels (Figure S5) after siRNAs treatment was previously reported for SMS1 [23].

### 2.5. SMS1 and 2OHOA Modulate the β-Catenin Pathway 

Since the interaction between N-Cadherin and β-catenin modulates the β-catenin signaling pathway [24,25], we investigated β-catenin response to 2OHOA treatment in the context of altered SMS expression. Abnormal activation of the Wnt/β-catenin pathway has been associated with GBM progression [26] and inhibition of the pathway by siRNA suppresses malignant glioma cell growth [27]. Upregulation of N-Cadherin is associated with inhibition of the β-catenin pathway, and when N-Cadherin/β-catenin complexes are cleaved at the plasma membrane, β-catenin is intensely phosphorylated at Thr41 and subsequently degraded [28]. β-catenin may be localized at the plasma membrane in association with N-Cadherin, in the cytoplasm where it is usually degraded, or in the nucleus, where it acts as a transcription factor to activate the expression of genes related to cell proliferation, such as AXIN2, Cyclin D1 or c-myc [29,30,31]. Here, we used AXIN2 mRNA expression as an indicator of β-catenin activity. Again, SMS1 and SMS2 displayed an inverse influence on this pathway (Figure 5H). Thus, activation of the β-catenin pathway was enhanced 82% when SMS1 was silenced, whereas either SMS1 overexpression or treatment with 2OHOA induced inhibition of this pathway (34% and 51%, respectively; Figure 5H). Conversely, SMS2 silencing or overexpression did not alter the activation of the β-catenin pathway (Figure 5H). In addition, the distribution of β-catenin protein was altered when SMS1 was overexpressed (Figure 5I), resulting in a clear cytoplasmic distribution and a strongly decreased presence in the nucleus.

### 2.6. SMS1 and SMS2 Modulate ER Stress but Only SMS1 Affects Autophagy

**Figure 6:**
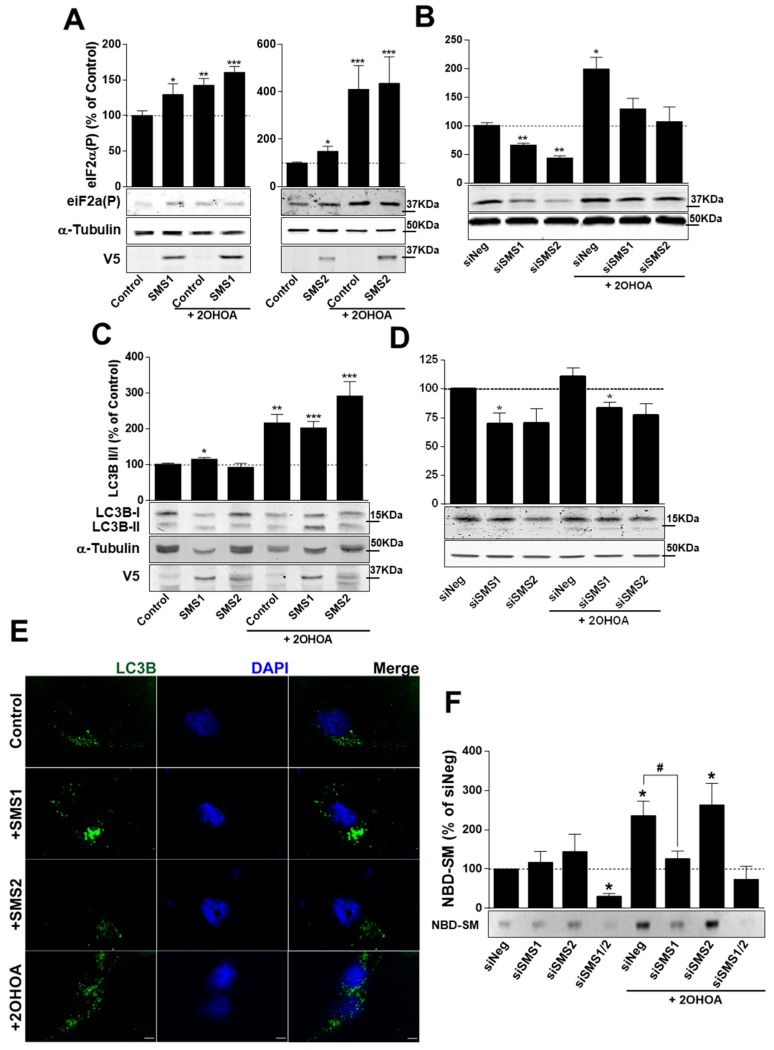
Effects of SMSs and 2OHOA on ER stress and Autophagy in U118 cells. Phospho-eIF2α [eIF2α(P): S51] levels in U118 cells, determined by immunoblotting, (**A**) after 72-h treatment with doxycycline (1 µg/mL) to induce SMS1 or SMS2 overexpression, 2OHOA (200 µM), or both, or (**B**) after treatment with siRNA targeting SMS1 or SMS2 for 48 h, with 2OHOA (200 µM) for 24 h, or both. Levels of LC3B II-to-LC3B I determined by immunoblotting in U118 cells (**C**) after 72-h treatment with doxycycline (1 µg/mL) to induce SMS1 or SMS2 overexpression, 2OHOA (200 µM), or both, or (**D**) after treatment with siRNA targeting SMS1 or SMS2 for 48 h, with 2OHOA (200 µM) for 24 h, or both. Untreated cells or cells not overexpressing SMSs were used as controls (100%), and the bars represent mean ± s.e.m values from 4–8 independent experiments; The differences were analyzed using a Student’s *t*-test or ANOVA *, *p* < 0.05; **, *p* < 0.01; ***, *p* < 0.001; # *p* < 0.05; between groups. SMS1 and SMS2 overexpression was measured using an anti-V5 antibody. (**E**) LC3B immunofluorescence (green) in U118 cells overexpressing SMS1, SMS2 or after 2OHOA treatment (200 µM) for 72 h. Nuclei were labeled with DAPI. Representative micrographies (single confocal plane) are shown. Scale bar, 5 µm. (**F**) NBD-SM synthesis after 3-h incubation with NBD-C6-Ceramide in U118 cells previously treated for 48 h with siRNAs (siNeg, siSMS1, siSMS2 or siSMS1+siSMS2) and in the presence or absence of 200 µM of 2OHOA (12 h).

2OHOA activates ER stress and macroautophagy in glioma cells [32,33]. An immediate response under ER stress is a general protein synthesis inhibition mechanism due to increased phosphorylation of the α subunit of the translation initiation factor eIF2α at Ser51 [34]. In turn, this effect mediates conversion of light chain 3 (LC3) from the soluble LC3-I to the membrane-bound LC3-II form, a key step in autophagy induction in mammalian cells [35]. These two ER stress markers (LC3 and eIF2α) were assessed and the levels of phosphorylated eIF2α increased after exposure to 2OHOA [32]. Overexpression of either SMS1 (Figure 6A, left) or SMS2 (Figure 6A, right) mimicked the increase in phospho-eIF2α induced by 2OHOA, whereas downregulation of either SMS1 or SMS2 decreased phosphorylation of eIF2α by 35% and 55%, respectively (Figure 6B). Furthermore, phosphorylation of eIF2α induced by 2OHOA was maintained following SMS1 or SMS2 overexpression (Figure 6A), although it was abolished when either SMS1 or SMS2 were silenced (Figure 6B). SMS1, but not SMS2, overexpression and 2OHOA provoked increase in the LC3B II-to-LC3B I ratio, (Figure 6C). Confocal microscopy revealed the cytoplasmic aggregation of LC3 upon SMS1 overexpression (Figure 6E). In contrast, LC3B II-to-LC3B I ratio decreased by 30% in response to SMS1 downregulation (Figure 6D). Furthermore, the 2-fold increase in LC3B II-to-LC3B I ratio induced by 2OHOA was maintained when SMS1 and SMS2 were overexpressed, and it was abolished when SMSs were silenced upon siRNA treatment.

### 2.7. 2OHOA Increases SMS Activity through SMS1 Exclusively

To determine if the overexpressed SMS1 and SMS2 proteins were enzymatically active, conversion of NBD-C6-Ceramide into NBD-C6-SM was measured. A clear increase in protein activity was observed when SMS1 or SMS2 were overexpressed (Figure S3), thus demonstrating that both overexpressed proteins were active.

The same activity assay was investigated when SMS1, SMS2 or both were silenced using specific siRNAs. Only when both SMSs were silenced total SMS activity was blocked, which suggested a compensatory mechanism whereby only one SMS is needed to maintain SM production. Interestingly, this compensatory activation would only rely on enzymatic activity since their expression was not significantly affected (Figure S5). In the presence of 2OHOA, the protein activity assay showed a clear increase of SMS activity, except when SMS1 or both SMSs were silenced. These results demonstrate that 2OHOA induction of SM synthesis requires SMS1, and further suggests that 2OHOA primarily interacts with SMS1 to increase SMS enzymatic activity (Figure 6F). 

## 3. Discussion

In the present study, we assessed the influence of SMS1 and SMS2 on glioma patients’ survival, cancer cell viability and responses to the antitumor drug, 2OHOA. We showed that either high SMS1 or low SMS2 mRNA levels represented a good prognostic factor in glioma. Moreover, the combination of SMS1-high plus SMS2-low expression resulted in a significant and marked increase in median survival compared to the other expression profiles. By contrast, SMS1-low and SMS2-high expression, independently or combined, was associated with poor prognosis and significantly worse survival parameters. In addition, we reported that the pharmacological effect of 2OHOA is due to the increase of SMS1 expression and enzymatic activity, and simultaneous reduction of SMS2 in glioma cells, which regulate cell growth and patient prognostic. SMS1 expression showed robust association with glioma patients’ survival as similar results were observed in 2 different databases, REMBRANDT (Figure 1) and GSE4412 (Figure 2).

The effect of SMSs overexpression on U118 GBM cells in vitro was in line with the clinical data that showed a decreased or increased in cell proliferation following SMS1 or SMS2 overexpression, respectively. For example, a reduced proliferation capacity in glioma cells overexpressing SMS1 would agree with longer survival in glioma patients with high SMS1 expression. However, when SMS1 and SMS2 were silenced using specific siRNAs, cell viability decreased in both cases, and this is consistent with the clinical response to low SMS2 levels, but not with the response to low SMS1 levels of expression. Nevertheless, this discrepancy may reflect the extremely efficient in vitro silencing of SMS1, which achieved an inhibition of expression stronger than any differences found in patients. Thus, although the lowest SMS1 expression was about 57%, the highest expression measured in glioma patients (Figure S8), siSMS1 caused a reduction to 10% of the control SMS1 expression in vitro, potentially compromising GBM cell survival. This premise is supported by the fact that SMS1 KO mice show deteriorated growth and male infertility, and they are born in a lower Mendelian ratio [36,37], which indicates the relevance of SMS1 expression during development.

These novel findings show the opposing roles of these two SMS isoforms in GBM cells and glioma patients, and that the antitumor drug 2OHOA, which has demonstrated safety and efficacy in phase I/IIA clinical trial, can differentially modulate SMS1, a potential new anticancer drug target. We also found a strong positive correlation between basal SMS1 mRNA levels and the pharmacological potency of 2OHOA based on IC_50_ values. These results suggest a direct interaction between 2OHOA and SMS1 and support the use of SMS1 basal expression as a prognostic biomarker to identify potential responders to 2OHOA treatment. This hypothesis is further supported by the facts that (1) only GBM cell lines responding to 2OHOA treatment increase SMS1 expression, and (2) the effect of increasing SMS activity by either SMS1 overexpression or 2OHOA treatment produced similar glioma cell growth inhibition. In fact, the lower SMS1 mRNA levels in human glioma cells relative to non-glioma cancer cells (both in vitro and in vivo) can explain the enhanced efficacy of 2OHOA against this type of cancer. Finally, the fact that induction of SM synthesis by 2OHOA was observed only in cells expressing SMS1 (independently of SMS2 expression) suggests a specific and direct interaction of 2OHOA with SMS1, particularly considering the short times of treatment needed to produce this increase in SMS activity. A recently published study questions the activation of SMS activity in response to treatment with 2OHOA [38]. However, substantial differences in the protocol used in this publication compared to previous studies [19] might well explain the divergent results obtained, such as the method of dissolution of the drug or the direct addition of the substrate NBD-Cer instead of adding it in the form of LUVs, among others. In this work [19], 2OHOA treatments, performed between 5 min and 24 h, indeed increased SMS activity, and now we provide further detail on this effect showing that SMS activity increase is exclusively due to the activation of SMS1 and that this effect fades away for longer incubation times.

GBM cell differentiation was assessed using N-Cadherin regulation as indicator, which was enhanced by SMS1, 2OHOA or both. SMS2 overexpression dampened SMS1 expression, which could in part explain the lack of effects of 2OHOA on N-Cadherin when SMS2 was overexpressed. Not only were the levels of N-Cadherin induced by 2OHOA relevant to cell differentiation, but its distribution was also relevant to the membrane recruitment of β-Catenin, thus driving its exclusion from the nucleus and its ensuing degradation [28]. The cellular localization of β-Catenin has a great influence on its activity: it is readily degraded in the cytoplasm, activation by Wnt induces its translocation to the nucleus and its subsequent participation in the expression of genes related to cell growth, or it may bind to transmembrane cadherins to participate in their association with actin cytoskeleton and cell differentiation. Here, it was shown that SMS1 overexpression was associated with both altered distribution of N-Cadherin and β-Catenin exclusion from the nucleus, all them molecular events involved in 2OHOA’s mechanism of action, that causes glioma cell cycle arrest, differentiation and death [3]. 

Overexpression of SMS1 but not SMS2 significantly activated macroautophagy via LC3 cleavage, which may explain the opposing roles of SMS1 and SMS2 in GBM. While autophagy is a cellular recycling mechanism, macroautophagy is a pivotal means to induce cancer cell death [30]. Most importantly, activation of autophagy can induce either cancer cell chemoresistance (when initiated via EGFR signaling for example) or autophagic cell death (e.g., when initiated by β-catenin, AKT or Ras signaling), depending on the tumor type and treatment. In fact, the activation of apoptosis and autophagy following knockdown of β-catenin was recently reported in head and neck squamous cancer cells [39,40]. In our in vitro GBM cell model, the mechanism of action by 2OHOA was associated with mislocalization of K-Ras [3]. Consistently with these results, similar K-Ras mislocalization was seen when sphingomyelinase was inhibited [41], which also increases SM levels. In addition, K-Ras mislocalization is also related to phosphatidylserine decrease in the plasma membrane [42] and 2OHOA produces this effect on U118 cells [19]. Here, it was shown that both 2OHOA treatment and overexpression of SMS1 dampened the activity of the β-catenin pathway, evident through the decrease in AXIN2 mRNA and compensatory increase by SMS1 silencing. Hence, 2OHOA-induced macroautophagy [32,33] appears to be mediated by β-catenin. In general, GBM cells in which the expression of SMS1 increased showed lower proliferative capacity (accompanied by reduced levels of DHFR), increased differentiation and induction of autophagic cell death. This effect was replicated or enhanced by 2OHOA, which in part explains its molecular mechanism of action.

Finally, we showed that only U118 cells expressing SMS1 underwent increased SM synthesis upon 2OHOA treatment. Our results demonstrate a direct modulation of SMS1 by 2OHOA, most likely due to direct interactions between these two molecular entities. The rapid response to treatment [19] and the ability of the SAM domain present only on SMS1 to bind to lipids [7] further support the hypothesis of a direct 2OHOA-SMS1 interaction. Thus, SMS1 activation induces glioma cell cycle arrest, differentiation and death. Because the main structural and functional difference between SMS1 and SMS2 is the SAM domain in SMS1, we hypothesize that this region could be involved in the divergent effects of 2OHOA on these isozymes. In addition to the SAM domain, the different cellular localization of SMS1 and SMS2, could explain their divergent effects on cell proliferation and glioma patient prognosis, as well as their differential response to 2OHOA treatment.

Notably, SMS1 silencing caused SMS2 mRNA increases, which might be associated with some of the effects observed after siSMS1 treatment. This situation reflects the importance of the balance between both isoforms and replicates the worst scenario for patients (Low-SMS1 in combination with High-SMS2). Nevertheless, SMS1 increase due to SMS2 silencing did not significantly increase the expression of the corresponding protein. This behavior has previously been reported for SMS1 [23], reflecting the high post-transcriptional regulation these enzymes undergo. SMS1 is a complex gene with several splicing forms [43] capable of generating circular and linear RNAs [44,45]. Furthermore, it contains recursive exons and participates in a multi-step splicing process involved in the generation of a wide array of RNAs, some of them with unknown roles [46]. 

Although neither SMS1 protein nor mRNA levels increased when SMS2 was silenced, its enzymatic activity was boosted, which suggests functional regulatory post-translational processes of this key enzyme. Alternatively, another enzymatically active SMS1 splicing isoform may be responsible for the increase in the enzymatic activity. The fact that only SMS1 silencing induced SMS2 mRNA increase, while SMS2 silencing did not affect SMS1 mRNA levels, reflected that most SM is synthesized by SMS1, so that its lack would induce a compensatory increase of SMS2 expression, whereas lack of SMS2 would not have a high impact in the cell. In favor of this, while SMS1 KO mice show male infertility and are born in a lower Mendelian ratio [36,37], SMS2 KO mice are seemingly healthy and only display reduced SM levels by about 20% [47]. The great complexity of the SMS1 gene and its strong post-transcriptional/translational regulation, suggest an important role in cells beyond its catalytic activity. 

Previous studies suggested that the effect of 2OHOA to increase SM levels was exerted on both SMS1 and SMS2 in the context of the state-of-the-art at that point in time [3,19]. However, more recent discoveries showing that both enzymes have ceramide phosphoethanolamine synthase activity [47] opened the possibility of a differential action of 2OHOA on these isozymes. The present work demonstrated that 2OHOA only regulated SMS1. SMS1 and SMS2 are important enzymes in glioma tumorigenesis, with value as prognostic biomarkers to patient survival and potential response to pharmacological treatment with 2OHOA. SMS1 and SMS2 appear to have opposite effects on GBM cell differentiation, proliferation and survival. Our results suggest that 2OHOA would be more efficacious in glioma/GBM (and possibly other cancer) patients with low SMS1 expression that augments upon treatment initiation. The present study elucidates unknown functions about membrane lipids in general and lipid enzymes in cancer cell proliferation and survival. It also sheds light on the mechanism of action of 2OHOA and, opens new avenues for the rational design of specific SMS2 inhibitors to treat GBM and potentially other cancers.

## 4. Materials and Methods 

### 4.1. Cancer Patient Data Collection and Processing

The potential clinical value of SMS1 and SMS2 gene expression was assessed in different datasets to investigate their correlation with the overall survival of patients with GBM and other cancer types. The “REpository for Molecular BRAin Neoplasia DaTa” [17] dataset was downloaded from NCI CA Integrator to evaluate the relationship between SMS1 and SMS2 expression and glioma patient survival. These data, which were used to produce Kaplan-Meier plots from the REMBRANDT dataset, were available at the Betastasis website (www.betastasis.com). The REMBRANDT database provides gene expression and survival data from 524 glioma patients, mainly grade III and IV (including 329 GBM patients).

The relationship between SMS1 gene expression and survival probability after diagnosis was also investigated in other cancer datasets using the PROGgeneV2 website tool http://watson.compbio.iupui.edu/chirayu/proggene/database/?url=proggene) [48]. The databases used to study this relationship were: GSE4412 for patients with grade III and IV glioma; TCGA-GBM for glioblastoma; GSE12417 for cytogenetically normal acute myeloid leukemia (CN-AML); GSE62254 for gastric cancer; TCGA-KIRK for kidney renal clear cell carcinoma (KIRC); GSE30219 for lung cancer; GSE4475 for lymphoma; GSE21501 for pancreatic ductal adenocarcinoma (PDAC); TCGA-SARC for sarcoma; and TCGA-SKCM for skin cutaneous melanoma (SKCM). For each dataset, cancer patient samples were classified into groups of high and low SMS1 expression with respect to the median expression.

The median survival and p-values from the log-rank tests were compared in Kaplan-Meier plots for low and high SMS expressing patient groups using the GraphPad Prism 6 or the PROGgeneV2 web tool, setting statistically significant difference between curves at *p* < 0.05. Overall survival at 2 and 5 years after diagnosis was calculated as indicated above, and presented as the mean ± standard error, showing the 95% upper and lower Confidence Intervals (CI). The correlation between SMS1 and SMS2 expression and lifespan is represented as boxes and whiskers, showing the 2.5–97.5 percentile data. The results were analyzed using a non-parametric Mann-Whitney test and were considered significant at *p* < 0.05 values (two tailed, unpaired data).

### 4.2. Cell Culture

GBM and other cancer cell lines were obtained from Apointech SI (Salamanca, Spain) and cultured in RPMI 1640 medium (Sigma-Aldrich, St Louis, MO, USA) supplemented with 10% Fetal Bovine Serum (FBS, Sigma-Aldrich), 100 units/mL penicillin and 100 μg/mL streptomycin (Labclinics, Barcelona, Spain). The cells were cultured in 2 mL of medium in 6-well plates for RNA extraction or in 4 mL of medium in P60 dishes for immunoblotting and incubated at 37 °C and in 5% CO_2_.

### 4.3. Cell Transfection and Protein Overexpression

Stably transfected cells were generated using the Tet-On^®^ 3G Inducible Expression System (Clontech, Madrid, Spain), according to the manufacturer’s instructions. G418 (200 ng/μL) and hygromycin (100 ng/μL) were used for maintenance of stably transfected cells. Doxycycline (1 µg/mL) was used to induce SMS1 or SMS2 overexpression. Lipofectamine 2000 (ThermoFisher, Barcelona, Spain) was used to achieve transient transfection following the manufacturer’s instructions.

### 4.4. Gene Expression Silencing

SMS1 and SMS2 gene expression silencing was performed by transient transfection of commercial siRNAs from Dharmacon, which were introduced in cells using RNAiMax (Life Technologies, Barcelona, Spain) followed by 48-h incubations. For siSMS1 silencing, the sense and antisense sequences used were GCCCAACUGCGAAGAAUAAUU and UUAUUCUUCGCAGUUGGGCUU, respectively (target sequence: GCCCAACTGCGAAGAATAA). For siSMS2 silencing, the sense and antisense sequences used were ACCGUCAUGAUCACAGUUGUAUU and UACAACUGUGAUCAUGACGGUUU, respectively (target sequence: ACCGTCATGATCACAGTTGTA). A negative siRNA control (siNeg) from Life Technologies was used (unknown mixed sequences).

### 4.5. Antibody Generation

Two newly generated tailor-made isoform-specific antibodies anti-SMS1 and anti-SMS2 were produced in rabbit (Proteogenix, Schiltigheim, France) using the following peptides, respectively: DIPTPDGSFSIKIKPNGMPNGY and PSDPTNTYARPAEPVEEENKNG. A complete characterization of these antibodies is showed in Figure S1.

### 4.6. Cell Lysis, Electrophoresis, Immunoblotting and Protein Quantification

Cells were washed with phosphate-buffered saline (PBS, pH 7.6) and scraped in 400 μL of protein extraction buffer (10 mM Tris-HCl, 2 mM EDTA, 1% SDS, 5 mM Protease Inhibitor Complete [ROCHE] and 1 mM Sodium Orthovanadate). Protein extract quantification and immunoblotting were performed as described in [32] with minimum variations. For standard curves (basal protein) increasing amounts of a standard protein extract (5, 15, 30 and 45 µg) were loaded on the same gel along with the experimental samples. The membranes were incubated overnight with primary antibodies diluted in fresh blocking solution: rabbit anti-SMS1 1:5000 or rabbit anti-SMS2 1:5000 from the above-described sera (ProteoGenix, Schiltigheim, France); mouse anti-N-Cadherin, anti-β-Catenin and anti-DHFR diluted 1:1000 (*BD-*Bioscience, Madrid, Spain); rabbit anti-LC3 and mouse anti-eIF2α diluted 1:1000 (Cell Signalling, Leiden, The Netherlands); mouse anti-V5 tag was diluted 1:5000 (Thermo Scientific, Barcelona, Spain). Antibody binding was detected with IRDye (800 CW) conjugated anti-mouse or anti-rabbit IgG (Li-cor, Biosciences, Lincoln, NE, USA) in blocking solution, incubating the membranes for 1 h at room temperature (1:10,000). Finally, protein content was quantified by integrated photodensitometry after near infrared scanning at 700 nm (Odissey, Li-cor Biosciences, Lincoln, NE, USA). α-Tubulin (1:10,000, Sigma) was assessed as a loading control. Bars in figures are representative of the average quantification from several experiments, each one with various replicates. The values in every lane were normalized to tubulin content and normalized values in treated cells were referred to those of control (untreated and not overexpressing) cells.

### 4.7. Immunofluorescence and Confocal Microscopy

Cells were seeded onto 25 mm circular coverslips in 24-well plates at a density of ca. 25 × 10^3^ cells/well in 1 mL of RPMI 1640 medium supplemented with 10% FBS. After incubation overnight, SMS1 or SMS2 cloned in pTRE3G (Clontech, Saint-Germain-en-Laye, France) was transiently expressed and overexpression was induced with doxycycline treatment for 48 h. 2OHOA treatment was maintained for 24 h. Cells were then fixed with 4% paraformaldehyde in phosphate buffer for 30 min at room temperature or with 100% methanol for 1 h at −20 °C, and they were subsequently washed with PBS.

Cells were incubated overnight at 4 °C with antibodies against LC3 (Cell Signalling, Leiden, The Netherlands), N-Cadherin or β-Catenin (BD-Bioscience, then washed 5 times with TBS and then incubated for 1 h at room temperature with the appropriate Alexa Fluor^®^ 488- or Alexa Fluor^®^ 598-conjugated IgGs (Invitrogen, Carlsbad, CA, USA). Finally, cells were washed 5 times with PBS, incubated with DAPI (Sigma, Darmstadt, Germany) for 5 min at room temperature and then washed twice with PBS. Coverslides were then mounted in Vectashield (Hardse: VECTOR) medium and examined under a confocal fluorescence microscope (Leica Microsystem TCS SPE) using a 63× objective lens.

### 4.8. RNA Isolation

Cells were seeded in 6-cm^2^ plates with RPMI 1640 medium supplemented with 10% FBS, 100 U/mL penicillin and 100 µg/mL streptomycin (PAA Laboratories GmbH, Austria). After 24 h, cells were exposed to 200 μM 2OHOA for 4, 8, 24 or 48 h. After treatment, the culture medium was removed, and the cells were washed with PBS. RNA was isolated using TriPure Isolation Reagent (a monophasic solution of phenol and guanidine thiocyanate at pH 4, Roche) according to the manufacturer’s instructions and it was quantified using a Nanodrop2000 Spectophotometer at 260 nm (Thermo Scientific).

### 4.9. Quantitative RT-PCR

The isolated RNA was reverse-transcribed using the High-Capacity cDNA Reverse Transcription Kit (Applied Biosystems, Barcelona, Spain). The target cDNA was amplified by Real-Time quantitative polymerase chain reaction (RTqPCR) using SYBR^®^ Premix Ex Taq™ II (Tli RNaseH Plus, Clontech, Saint-Germain-en-Laye, France) and ROX Reference Dye (50×) in a Step One Plus Real Time PCR thermal cycler (Applied Biosystems). The primers used were:

SMS1 Forward (Fw): TGACTCCAGTGCAACGTGAC
SMS1 Reverse (Rv): GTCCACACTCCTTCAGTCGC
SMS2 Fw: TTAATCTGCTGGCTGCTGAG
SMS2 Rv: ACCAATCTTCTGAACCCGTG
AXIN2 Fw: GAGTGGACTTGTGCCGACTTCA
AXIN2 Rv: GGTGGCGTTGCAAAGACATAG
HPRT Fw: TGACCTTGATTTATTTTGCATACC
HPRT Rv: CGAGCAAGACGTTCAGTCCT

A standard curve of known cDNA concentrations was used to quantify the mRNA levels using duplicate serial dilutions starting from 50 ng of cDNA. 

A Step One Plus Real Time PCR thermal cycler (Applied Biosystem) was used and the relative mRNA content was calculated using StepOne Software v2.3, expressed in arbitrary units (a.u.) using standard curves with different amounts of cDNA.

### 4.10. Enzymatic Assay

After overexpression or silencing and treatment in the presence or absence of 2OHOA, 3 µM of NBD-Ceramide was added to cells in culture and incubated for 3 h. Then, cells were scraped in a proper volume of extraction buffer (10 mM Tris-HCl, 2 mM EDTA and Protease Inhibitor Complete, ROCHE). After homogenization with ultrasounds, 200 µg of total protein diluted in a final volume of 250 µL of extraction buffer were mixed with 1.25 mL of chloroform:methanol (2:1) to extract lipids. The organic phase was collected after centrifugation for 10 min at 1000× *g* and solvents were evaporated under N_2_ flux.

### 4.11. Thin Layer Chromatography (TLC)

Lipids extracted as indicated above were dissolved in chloroform and loaded on 20 × 20 cm silica plates. Lipid species were resolved using a mobile phase of Chloroform:Methanol:Water:Acetic acid (60:50:1:4 v:v:v:v). Plates were scanned on a fluorescent Bio-Rad (FX) scanner. NBD-SM synthesis was quantified by integrated optical density analysis.

### 4.12. Data Analysis

For Kaplan-Meier plots, the log-rank test was used to define differences between high and low SMS expression populations. For in vitro studies, data were expressed as the mean ± SEM or the % relative to untreated controls from the number of independent experiments indicated, and the differences were analyzed using a Student’s *t*-test or ANOVA. The correlations between IC_50_ values and protein levels were analyzed using a Pearson’s correlation test. Differences were considered significant at *p* < 0.05.

## 5. Conclusions

The present results suggest that SMS1 and SMS2 had opposite effects on glioma patients’ survival, glioma cell growth and response to 2OHOA treatment. SMSs signature could constitute a valuable prognostic biomarker, high SMS1 and low SMS2 indicating a better disease prognosis. Additionally, low basal SMS1 mRNA levels predict positive response to 2OHOA in GBM cell lines. Regarding 2OHOA’s mechanism of action, we demonstrated that 2OHOA induction of SM synthesis is due exclusively to SMS1 activation, and not to SMS2 activation.

## Figures and Tables

**Table 1:** Patients at risk from Kaplan-Meier plots in Figure 2. Patients were censored when they were alive at the time of study; mo.: months; n: number of patients; CI: confidence interval.

Cancer Type	Data Set	SMS1 Express	n Total/Censored	Median Survival (mo.)	2-Year				5-Year			
					*n* at Risk	Survival (%)	Lower 95%CI	Upper 95%CI	*n* at Risk	Survival (%)	Lower 95%CI	Upper 95%CI
Glioma	GSE4412	High	42/18	23.87	22	49.8	36.7	67.5	N/A	N/A	N/A	N/A
		Low	41/8	9.93	6	15.4	7.0	33.8	N/A	N/A	N/A	N/A
GMB	TCGA-GMB	High	271/60	14.83	58	27.9	22.6	34.6	13	9.9	6.3	15.6
		Low	271/56	12.95	36	19.4	14.7	25.7	5	2.3	0.9	6.0
CN-AML	GSE12417	High	82/30	11.18	26	37.5	28	50.1	N/A	N/A	N/A	N/A
		Low	81/30	8.42	26	35.9	26.4	48.8	N/A	N/A	N/A	N/A
Gastric	GSE62254	High	150/90	N/A	116	76.7	70.2	83.7	97	64.0	56.8	72.2
		Low	149/58	38.99	91	60.4	53	68.8	59	40.9	33.7	49.6
KIRC	TCGA-KIRC	High	117/93	93.04	71	84.9	77.8	92.6	41	76.8	67.87	87.0
		Low	117/74	56.35	66	80.9	73.3	89.4	24	48.4	37.8	62.0
Lung	GSE30219	High	141/60	83.84	111	77.0	70.3	84.3	69	58.1	50.3	67.1
		Low	141/37	28.60	77	53.8	46.2	62.7	49	37.8	30.52	46.8
Lymphoma	GSE4475	High	79/46	115.00	44	66.0	56.0	77.9	23	53.4	42.5	67.3
		Low	79/37	33.44	35	53.4	43.0	66.3	19	41.4	30.7	55.9
PDAC	GSE21501	High	51/22	21.70	17	45.3	32.3	63.5	N/A	N/A	N/A	N/A
		Low	51/14	14.79	11	22.0	12.4	38.9	N/A	N/A	N/A	N/A
Sarcoma	TCGA-SACR	High	117/86	81.01	59	77.7	69.2	87.2	16	57.5	45.2	73.1
		Low	117/76	53.49	50	75.0	66.2	84.9	18	43.9	32.3	59.7
SKCM	TCGA-SKMC	High	82/46	167.87	67	88.3	81.4	95.8	41	69.0	58.9	80.8
		Low	81/41	48.59	47	72.9	63	84.5	21	47.1	35.7	62.1

## Supplementary Materials

The following are available online at http://www.mdpi.com/2072-6694/11/1/88/s1, Figure S1: SMS1 and SMS2 protein sequences alignment, Figure S2: Characterization of newly generated SMS1 and SMS2 antibodies, Figure S3: Characterization of overexpressed SMS1 and SMS2 proteins, Figure S4: Human SF295 GBM cell viability, Figure S5: Cell viability after incubation with siRNA against SMS1 or SMS2, Figure S6: Representative immunoblots showing changes in SMS1 or SMS2 protein levels, Figure S7: DHFR protein levels in SF295 and U251 cells overexpressing SMS2, Figure S8: SMS1 and SMS2 gene expression in GBM patient samples, Table S1: IC_50_ values of different cancer cell lines after a 72 h exposure to 2OHOA, Table S2: Changes in SMS1-to-SMS2 protein ratio in response to 2OHOA treatment and IC_50_ for 2OHOA, Table S3: Basal mRNA levels (arbitrary units, a.u.) in several GBM cell lines and non-tumor (MRC5) cells.
