# Supplementary material for: The Opposing Contribution of SMS1 and SMS2 to Glioma Progression and Their Value in the Therapeutic Response to 2OHOA
Source: Cancers (Basel). 2019 Jan 14;11(1):88. doi: 10.3390/cancers11010088 (PMC6356341; doi:10.3390/cancers11010088)
Supplement: Supplementary file 1 [file cancers-11-00088-s001.pdf]

Supplementary Materials: The Opposing Contribution of SMS1 and SMS2 to Glioma Progression and Their Value in the Therapeutic Response to 2OHOA

Paula Fernández-García, Catalina A. Rosselló, Raquel Rodríguez-Lorca, Roberto Beteta-Göbel, Javier Fernández-Díaz, Victoria Lladó, Xavier Busquets and Pablo V. Escribá

```
CLUSTAL O(1.2.4) multiple sequence alignment

sp|Q86VZ5|SMS1_HUMAN      MKEVVYWSPKKVADWLLENAMPEYCEPLEHFTGQDLINLTQEDFKKPPLCRVSSDNGQRL 60
sp|Q8NHU3|SMS2_HUMAN      ------------------------------------------------------------ 0

sp|Q86VZ5|SMS1_HUMAN      LDMIETLKMEHHLEAHKNGHANGHLNIGVDIPTPDGSFSIKIKPNGMPNGYR------KE 114
sp|Q8NHU3|SMS2_HUMAN      MDIIETAKLEEHLENQPSDPTNTYARPAE--PVEEENKNGNGKPKSLSSGLRKGTKKYPD 58
                          :*:*** *:*.*** :  .. :* :  .  .     *. :  .  .  : **:.: .* *      :

sp|Q86VZ5|SMS1_HUMAN      MIKIPMPELERSQYPMEWGKTFLAFLYALSCFVLTTVMISVVHERVPPKEVQPPLPDTFF 174
sp|Q8NHU3|SMS2_HUMAN      YIQIAMPTESRNKFPLEWWKTGIAFIYAVFNLVLTTVMITVVHERVPPKELSPPLPDKFF 118
                           *:* **  .*.::*:** ** :**:**:  :*******:**********:.*****.**

sp|Q86VZ5|SMS1_HUMAN      DHFNRVQWAFSICEINGMILVGLWLIQWLLLKYKSIISRRFFCIVGTLYLYRCITMYVTT 234
sp|Q8NHU3|SMS2_HUMAN      DYIDRVKWAFSVSEINGIILVGLWITQWLFLRYKSIVGRRFCFIIGTLYLYRCITMYVTT 178
                          *:::**:****:.****:******: ***:*:****:.***  *:***************

sp|Q86VZ5|SMS1_HUMAN      LPVPGMHFNCSPKLFGDWEAQLRRIMKLIAGGGLSITGSHNMCGDYLYSGHTVMLTLTYL 294
sp|Q8NHU3|SMS2_HUMAN      LPVPGMHFQCAPKLNGDSQAKVQRILRLISGGGLSITGSHILCGDFLFSGHTVTLTLTYL 238
                          ********:*:*** ** :*:::**::**:**********  :***:*:***** ******

sp|Q86VZ5|SMS1_HUMAN      FIKEYSPRRLWWYHWICWLLSVVGIFCILLAHDHYTVDVVVAYYITTRLFWWYHTMANQQ 354
sp|Q8NHU3|SMS2_HUMAN      FIKEYSPRHFWWYHLICWLLSAAGIICILVAHEHYTIDVIIAYYITTRLFWWYHSMANEK 298
                          ********::**** ******..**:***:**:***:**::**************:***::

sp|Q86VZ5|SMS1_HUMAN      VLKEASQMNLLARVWWYRPFQYFEKNVQGIVPRSYHWPFPWPVVHLSRQV-KYSRLVNDT 413
sp|Q8NHU3|SMS2_HUMAN      NLKVSSQTNFLSRAWWFPIFYFFEKNVQGSIPCCFSWPLSWPPGCFKSSCKKYSRVQKIG 358
                           ** :** *:*:*.**:   * :******* :*  .: **: **     :. .  ****: :

sp|Q86VZ5|SMS1_HUMAN      ------- 413
sp|Q8NHU3|SMS2_HUMAN      EDNEKST 365
```

Figure S1. SMS1 and SMS2 protein sequences alignment. * Identical amino acid; :/. Homolog amino acid; bold: SAM domain; italic: transmembrane domain.

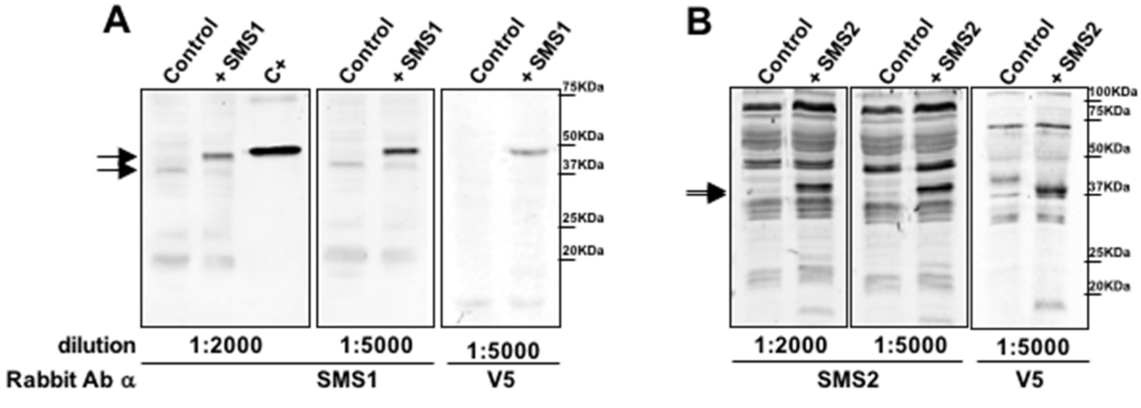

Figure S2. Characterization of new SMS1(**A**) and SMS2 (**B**) antibodies generated in rabbit after inducing overexpression. Both endogenous (Control) and overexpressed (+SMS1 or +SMS2) proteins were detected using anti-SMS (bottom label SMS1 or SMS2; total protein) or anti-V5 (bottom label V5: overexpressed protein). Immunoreactive bands for SMSs or V5-containing SMSs showed identical electrophoretic mobility, demonstrating specificity of anti-SMS antisera. Other immunoreactive bands could correspond to peptides produced after alternative splicing or oligomeric enzyme forms. SMS antibodies also detected purified SMSs produced in Sf9 cells, which further confirmed the specificity of the antibody (C+).

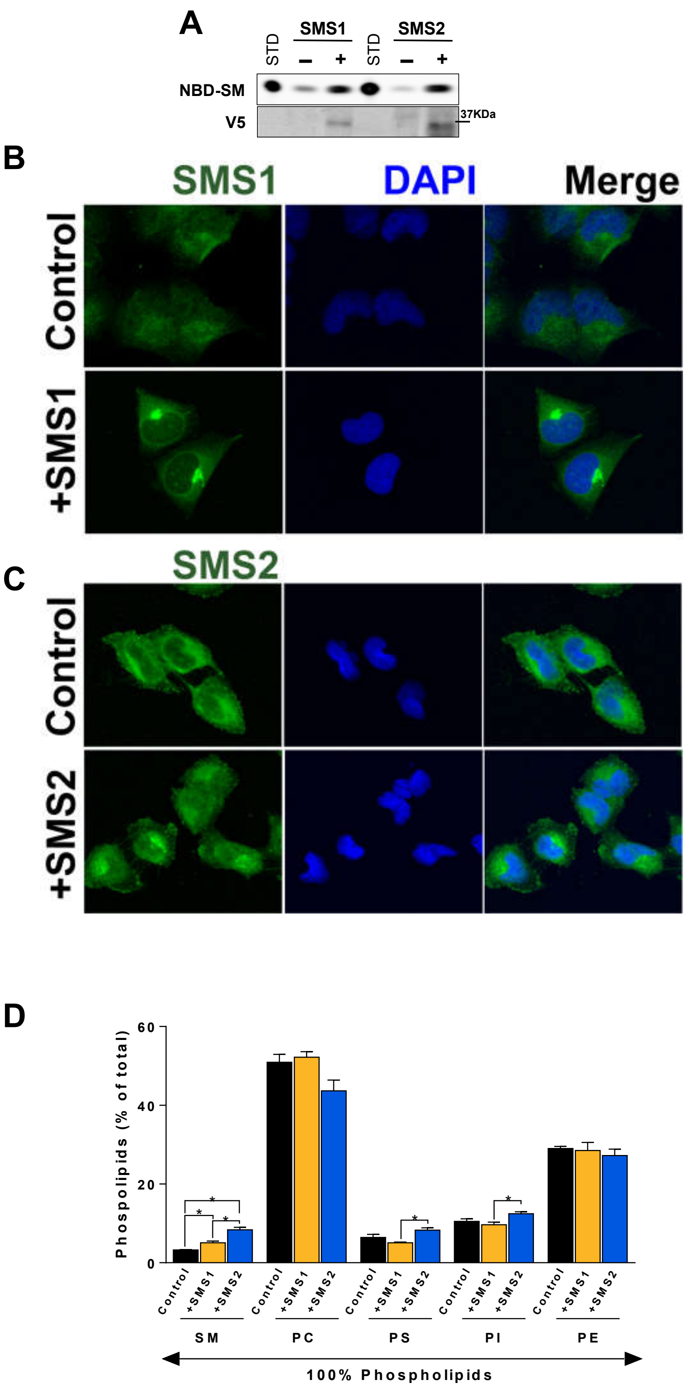

Figure S3. Characterization of overexpressed SMS1 and SMS2 proteins. (**A**) SMS activity assay. Lipids were fractioned by Thin Layer Chromatography (TLC) and newly synthesized NBD-C6-SM was quantified by fluorescence image analysis in control U118 cells (−) or U118 cells overexpressing (+) SMS. Immunofluorescence microscopy images showing the localization and density of endogenous (Control) or overexpressed SMS1 (**B**) or SMS2 (**C**) proteins. Images were acquired using same parameters as controls. D: Quantification of the main phospholipid species after SMS1 (yellow) or SMS2 (blue) overexpression (Sphingomyelin (SM), Phosphatidylcholine (PC), Phosphatidylserine (PS), Phosphatidylinositol (PI) and Phosphatidylethanolamine (PE)). Shown are the means ± SEM of at least three independent experiments. * $p < 0.05$.

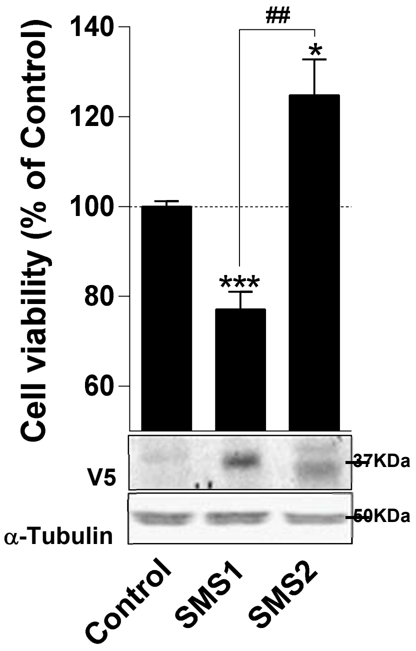

Figure S4. Human SF295 GBM cell viability after 48 h of SMS1 or SMS2 overexpression (transient transfection) relative to non-overexpressing control cells. This result confirms that shown in Figure 4A (U118 cells) but in a different cell line. Shown are the means ± SEM of at least three independent experiments. * $p < 0.05$, *** $p < 0.001$ versus control. ## $p < 0.01$.

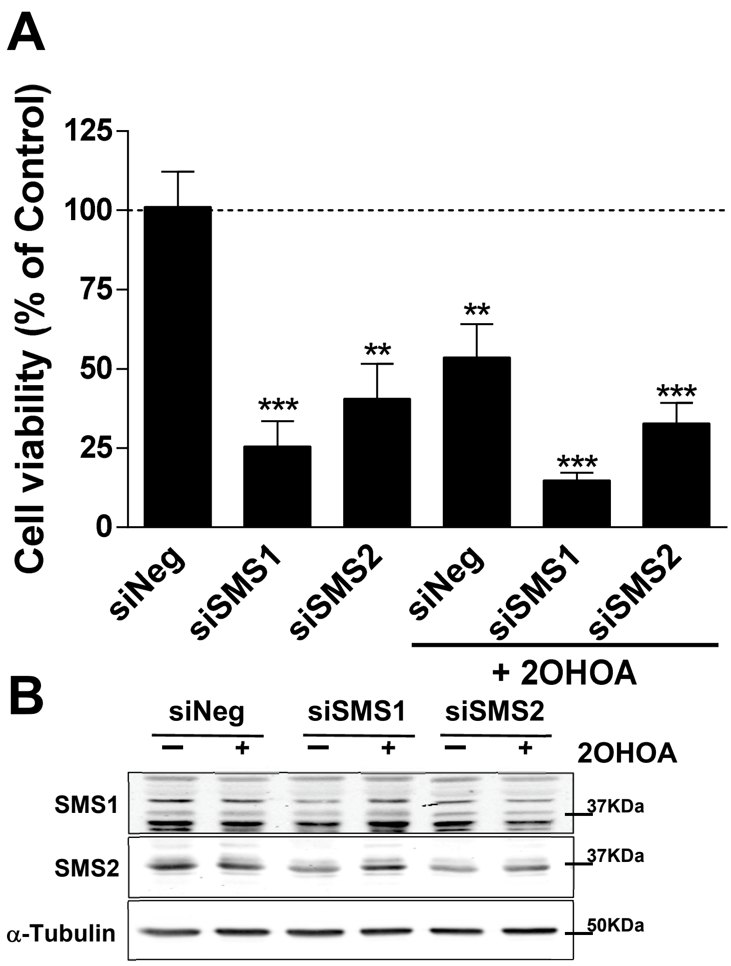

Figure S5. (**A**) Cell viability after incubation with siRNA against SMS1 or SMS2 in presence or absence for 2OHOA (200 μM) for 48 h. Negative siRNA was used as control. Shown are the means ± SEM of at least three independent experiments. * $p < 0.05$, ** $p < 0.01$, *** $p < 0.001$ versus control. (**B**) Representative immunoblots showing silencing efficacy.

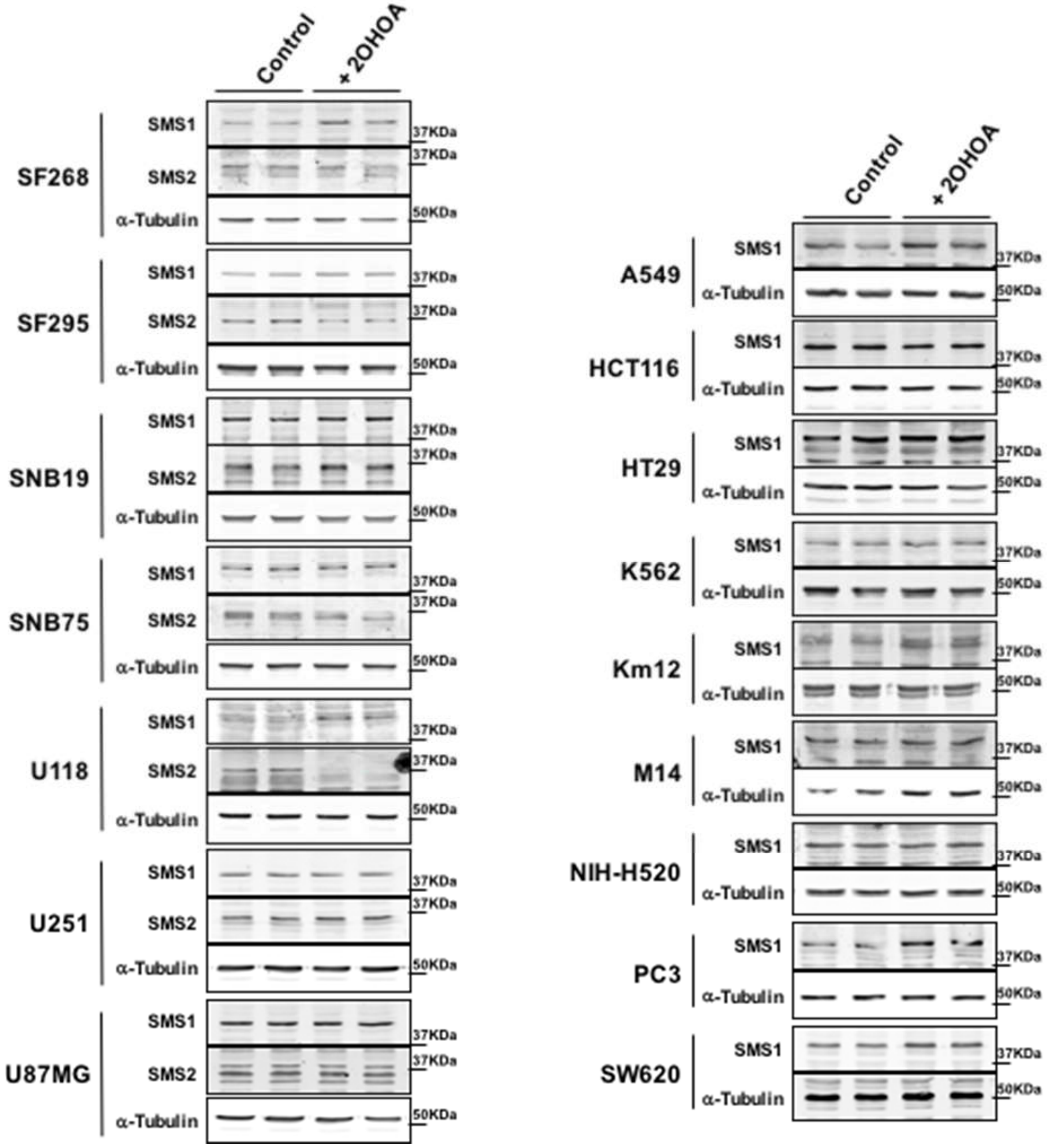

Figure S6. Representative immunoblots showing changes in SMS1 or SMS2 protein levels after 48-h treatment with 2OHOA in several cancer cell lines. Blots on the left correspond to human GBM cell lines (SF268, SF295, SNB19, SNB75, U118, U251 and U87MG). Blots on the right correspond to different human cancer cell lines (non-small cells lung cancer: A549 and NIH-H520, colon: HCT116, HT29, KM12 and SW620, leukemia: K562, melanoma: M14 and prostate: PC3).

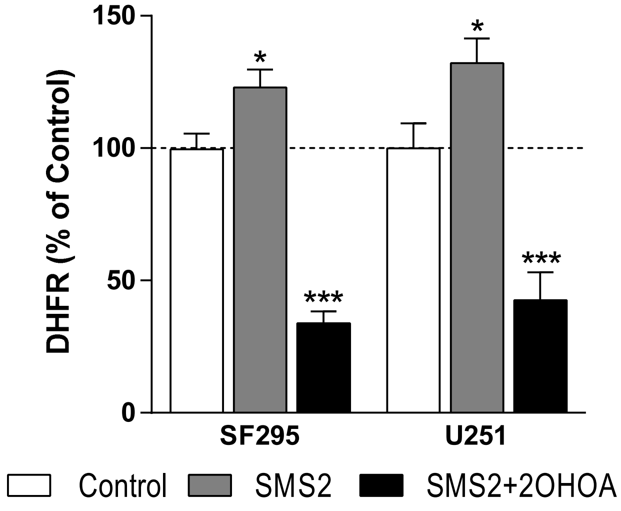

Figure S7. DHFR protein levels in SF295 and U251 cells overexpressing SMS2 (48-h transient transfection) in the presence or absence (Control) of 200 μM 2OHOA. Shown are the means ± SEM of at least three independent experiments. * $p < 0.05$, *** $p < 0.001$ versus control.

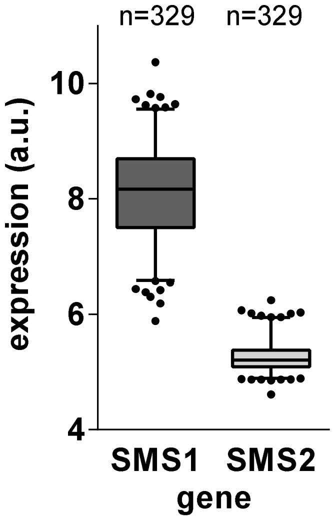

Figure S8. SMS1 and SMS2 gene expression in GBM patient samples (data from the REMBRANDT database; a.u.: arbitrary units).

Table S1. IC_{50} values of different cancer cell lines after a 72 h exposure to 2OHOA.

Cell line	IC_{50} (μM)	Tumour	Cell line	IC_{50} (μM)	Tumour
Km12	89.13	Colon	SF295	300	GBM
A549	90.82	Lung	NCIH520	342.97	Lung
U118	200	GBM	M14	348.8	Melanoma
PC3	203.74	Prostate	HCT116	418.47	Colon
K562	208.59	Leukemia	U87MG	420	GBM
SF268	240	GBM	SNB19	>600	GBM
HT29	271.1	Colon	SNB75	>600	GBM
SW620	284.13	Colon			

Table S2. Changes in SMS1-to-SMS2 protein ratio in response to 2OHOA treatment and IC_{50} for 2OHOA in several human GBM cell lines (data from Figure 3B).

Cell line	SMS1/SMS2 ± S.E.M.	IC_{50} (μM)
U118	1.642 ±0.13	200
SF268	1.625 ±0.13	240
SF295	1.757 ±0.28	300
U251	1.180 ±0.06	426.5
SNB19	0.715 ±0.08	>600
SNB75	1.046 ±0.09	>600

Table S3. Basal mRNA levels (arbitrary units, a.u.) in several GBM cell lines and non-tumor (MRC5) cells.

Cell line	Basal mRNA (a.u.)
U118	0.24
SF268	0.44
SF295	0.87
U251	1.20
SNB19	1.88
SNB75	2.01
U87-MG	4.45
MRC-5	7.58
